# MicroRNAs in the aqueous humor of patients with different types of glaucoma

**DOI:** 10.1007/s00417-021-05214-z

**Published:** 2021-04-30

**Authors:** Ewa Kosior-Jarecka, Marcin Czop, Karolina Gasińska, Dominika Wróbel-Dudzińska, Daniel P. Zalewski, Anna Bogucka-Kocka, Janusz Kocki, Tomasz Żarnowski

**Affiliations:** 1 Department of Diagnostics and Microsurgery of Glaucoma, Medical University of Lublin, ul. Chmielna 1, 20-079 Lublin, Poland; 2 Department of Clinical Genetics, Medical University of Lublin, ul. Radziwiłłowska 11, 20-080 Lublin, Poland; 3 Department of Biology and Genetics, Medical University of Lublin, ul. Chodźki 4a, 20-093 Lublin, Poland

**Keywords:** MicroRNA, Aqueous humor, Glaucoma, POAG, PACG, PEXG

## Abstract

**Purpose:**

The aim of the study was to compare the frequency and the level of expression of selected miRNAs in the aqueous humor of patients with various types of glaucoma.

**Methods:**

The studied group consisted of 42 patients with glaucoma: 19 with primary open-angle glaucoma (POAG), 14 with pseudoexfoliation glaucoma (PEXG), 9 with primary angle closure glaucoma (PACG), and the control group of 36 patients with senile cataract without glaucoma. The real-time polymerase chain reaction method was used to analyze the expression of miRNAs.

**Results:**

There were no significant differences in the frequency and the level of miRNA expression between various types of glaucoma. There was a tendency for hsa-miR-6722-3p and hsa-miR-184 to be expressed more frequently in PEXG and hsa-miR-1260b in POAG. The expression levels of hsa-miR-1260b and hsa-miR-6515-3p were correlated with age in POAG. Target annotation and functional analyses showed that genes targeted by the most frequently expressed miRNAs (hsa-miR-1202, -1260b, -184, -187-5p, -6515-3p, -6722-3p, and hsa-mir-4634) are involved mainly in response to hypoxia, cardiovascular system development, and apoptosis.

**Conclusion:**

Hsa-miR-1260b was the most abundantly expressed among studied miRNAs and may be a potential biomarker of clinical status in PEXG and PACG.

**Supplementary Information:**

The online version contains supplementary material available at 10.1007/s00417-021-05214-z.


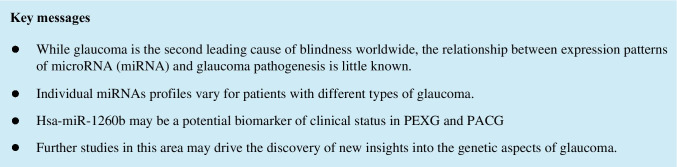


## Introduction

Glaucoma is a multifactorial disease involving retinal ganglion cells and has been estimated by WHO as the second leading cause of blindness worldwide [1]. The main risk factor for glaucoma is increase in intraocular pressure (IOP) caused by decreased outflow of aqueous humour (AH) from the anterior chamber [2]. Glaucoma is a group of optic neuropathies with similar morphology of the visual field defect and optic disc appearance. However, the mechanisms underlying the disease might be various. In most cases, the disease is connected with elevated IOP, but there are also cases with continuous progression despite low IOP values. Additionally, the mechanisms causing IOP elevation are also different in glaucoma types. MiRNAs are small, noncoding RNA molecules involved in RNA silencing and regulation of gene expression at the post-transcriptional level. MiRNAs predominantly act to reduce target gene expression [3]. Expression of miRNAs is often typical for a particular tissue or during essential cellular processes [4, 5]. MiRNAs act intracellularly but have also been detected in most body fluids, where they are preserved in microvesicles, exosomes, or bound to carrier proteins, providing a remarkable stability of miRNA [6]. A lot of extracellular miRNAs from biofluids has been identified as biomarkers for cancer, cardiovascular diseases, diabetes, ocular diseases, and many other disorders [4, 7–14]. Ocular fluids (tears, AH and vitreous humour) also have been reported to contain extracellular miRNAs [4, 15–18]. Alterations in levels of AH components, including cells, proteins, and miRNAs, may reflect the pathogenic process underlying the increase in IOP during the course of glaucoma. Only a few studies investigated miRNA expression in AH of glaucoma patients [4, 6, 15].Therefore, it is interesting whether the expression patterns of microRNA (miRNA) may reflect this variety. Tanaka et al. [19] showed that individual miRNA profiles vary for patients with glaucoma and the number of commonly detected miRNAs was limited, but combining these markers had the potential to increase the sensitivity of glaucoma diagnosis and to predict stage of advancement [4, 19], which is not possible with genome analysis [19].

The aim of the study was to compare the frequency and the levels of selected miRNAs expression in AH of patients with various types of glaucoma: primary open angle glaucoma (POAG), pseudoexfoliation glaucoma (PEXG), and primary angle closure glaucoma (PACG) as well as to identify miRNA-dependent mechanisms contributing to these types of glaucoma.

## Materials and methods

The study was performed in accordance with the tenets of the Declaration of Helsinki and the study design was approved by the Ethics Committee at the Medical University of Lublin (approval No. KE-0254/107/2020). The studied group consisted of 42 patients with glaucoma (19 POAG, 14 PEXG, and 9 PACG) and 36 control patients with senile cataract without glaucoma nor elevated IOP. Informed consent was signed by all participants before enrolment to the study. Patients’ demographic and clinical data are presented in Table 1.

**Table 1:** Demographic and clinical characteristics of the studied groups

Feature	Studied groups				Statistical analysis
	PACG (*n* = 9)	POAG (*n* = 19)	PEXG (*n* = 14)	Cataract (*n* = 36)	
Gender:					
Male	6	8	6	17	*p* > 0.05
Female	3	11	8	19	
Age^1^	75.00 ± 8.40 ^a^	72.11 ± 7.14 ^a^	79.21 ± 4.37 ^ab^	77.74 ± 8.65 ^b^	*p* ≤ 0.001
Maximum IOP^1^	35.56 ± 9.74 ^ab^	34.16 ± 13.79 ^a^	24.50 ± 7.49 ^b^	18.28 ± 2.76 ^ab^	*p* ≤ 0.05
MD^1^	− 16.24 ± 9.97 ^a^	− 14.23 ± 10.27 ^a^	− 12.75 ± 8.51 ^a^	0.16 ± 096	*p* > 0.05

^1^Mean ± standard deviation (SD). *IOP* intraocular pressure, *MD* mean defect. Groups in row not sharing the same letter are statistically different at *p* ≤ 0.05. *POAG* primary open angle glaucoma, *PEXG* pseudoexfoliation glaucoma, *PACG* primary angle closure glaucoma

All participants underwent planned cataract surgery at the Department of Diagnostics and Microsurgery of Glaucoma, Medical University of Lublin, Poland.

Before the surgery, all patients underwent detailed ophthalmic examination, included best corrected visual acuity (BCVA) assessment (using Snellen charts), slit-lamp biomicroscopy, gonioscopy, ultrasonic central corneal thickness measurements, IOP with Goldmann applanation tonometry, and stereoscopic optic nerve head (ONH) examination as well as a detailed medical history analysis. Detailed inclusion criteria were as follows:

1. Signed informed consent
2. The age of over 18 years old
3. Glaucomatous neuropathy
4. BCVA better than 0.1 (measured using Snellen charts)
5. Senile cataract with BCVA worse than 0.6
6. No ocular diseases except for cataract and glaucoma
7. No previous intraocular surgeries (except for laser iridotomy in PACG patients)
8. No diabetes mellitus

Patients with glaucoma additionally had visual field examination (24–2 strategy on Humphrey Perimeter). Glaucoma was diagnosed based upon the clinical determination of glaucomatous ONH damage (localised or diffuse neuroretinal rim thinning, rim notching, excavation, and/or retinal nerve fiber layer defect) associated with typical, reproducible standard automated perimetry defects. Glaucomatous defect on standard automated perimetry was defined based upon a glaucoma hemifield test result outside normal limits and the presence of at least 3 contiguous test points within the same hemifield on the pattern deviation plot at *P* < 1%, with at least 1 point at *P* < 0.5%, on at least 2 consecutive tests, with reliability indices better than 15%.

POAG was diagnosed when wide open-angle (grade III/IV according to Schaffer’s classification) was observed on gonioscopy without the features of any secondary glaucoma. PACG was diagnosed in patients with documented primary angle closure in their medical record who had undergone laser peripheral iridotomy. PEXG was diagnosed when glaucomatous optic neuropathy was accompanied by the presence of dandruff-like pseudoexfoliation material on the anterior lens capsule.

AH samples (~ 100 μl) were obtained from the anterior chamber by an experienced surgeon (TZ) at the beginning of the cataract surgery with a special care to avoid contamination with blood or tears. MiRNA was immediately isolated from AH samples using TaqMan MicroRNA Cells-to-CT Kit (Ambion, Austin, TX, USA) according to the manufacturer’s instructions. The isolated miRNA was stored at − 80 °C for further analysis.

The absorbance of miRNA was measured using a NanoDrop 2000c spectrophotometer (Thermo Fisher Scientific, Waltham, MA, USA), which allowed the authors to perform qualitative and quantitative evaluation. The amount of miRNA per sample in all cases was in the range of 1–350 ng. In addition, RNA analysis was performed using the Agilent Bianalyzer 2100 (Agilent Technologies, Lithuania) and Pico RNA Kit according to the manufacturer’s procedure (Supplementary Fig. 1).

Twenty-two miRNAs were selected for analysis on the basis of previous publications [19, 20], which showed them to be the most abundant in AH. The list of selected miRNAs was provided in Table 2.

**Table 2:** List of miRNAs examined in the study

Assay ID	Assay name	miRBase ID ^1^	miRBase Accesion Number ^1^	Mature miRNA Sequence ^1^
464340_mat	hsa-miR-4433	hsa-miR-4433a-3p	MIMAT0018949	ACAGGAGUGGGGGUGGGACAU
002,362	hsa-miR-202*	hsa-miR-202-5p	MIMAT0002810	UUCCUAUGCAUAUACUUCUUUG
463784_mat	hsa-miR-4725-3p	hsa-miR-4725-3p	MIMAT0019844	UGGGGAAGGCGUCAGUGUCGGG
002,404	hsa-let-7b*	hsa-let-7b-3p	MIMAT0004482	CUAUACAACCUACUGCCUUCCC
000,485	hsa-miR-184	hsa-miR-184	MIMAT0000454	UGGACGGAGAACUGAUAAGGGU
002,858	hsa-miR-1202	hsa-miR-1202	MIMAT0005865	GUGCCAGCUGCAGUGGGGGAG
000,399	hsa-miR-23a	hsa-miR-23a-3p	MIMAT0000078	AUCACAUUGCCAGGGAUUUCC
465775_mat	hsa-miR-3663-3p	hsa-miR-3663-3p	MIMAT0018085	UGAGCACCACACAGGCCGGGCGC
474380_mat	hsa-miR-6722-3p	hsa-miR-6722-3p	MIMAT0025854	UGCAGGGGUCGGGUGGGCCAGG
000,449	hsa-miR-125b	hsa-miR-125b-5p	MIMAT0000423	UCCCUGAGACCCUAACUUGUGA
471487_mat	hsa-miR-6515-3p	hsa-miR-6515-3p	MIMAT0025487	UCUCUUCAUCUACCCCCCAG
002,740	hsa-miR-187*	hsa-miR-187-5p	MIMAT0004561	GGCUACAACACAGGACCCGGGC
242525_mat	hsa-miR-1260b	hsa-miR-1260b	MIMAT0015041	AUCCCACCACUGCCACCAU
242546_mat	hsa-miR-3197	hsa-miR-3197	MIMAT0015082	GGAGGCGCAGGCUCGGAAAGGCG
476356_mat	hsa-miR-5001-5p	hsa-miR-5001-5p	MIMAT0021021	AGGGCUGGACUCAGCGGCGGAGCU
476806_mat	hsa-miR-6132	hsa-miR-6132	MIMAT0024616	AGCAGGGCUGGGGAUUGCAG
463820_mat	hsa-miR-4749-5p	hsa-miR-4749-5p	MIMAT0019885	UGCGGGGACAGGCCAGGGCAUC
475810_mat	hsa-miR-6717-5p	hsa-miR-6717-5p	MIMAT0025846	AGGCGAUGUGGGGAUGUAGAGA
462391_mat	hsa-miR-3940-5p	hsa-miR-3940-5p	MIMAT0019229	GUGGGUUGGGGCGGGCUCUG
464264_mat	hsa-miR-4484	hsa-miR-4484	MIMAT0019018	AAAAGGCGGGAGAAGCCCCA
463950_mat	hsa-miR-4467	hsa-miR-4467	MIMAT0018994	UGGCGGCGGUAGUUAUGGGCUU
Hs04258334_pri	hsa-mir-4634	hsa-mir-4634	MI0017261 (stem loop sequence)	GGACAAGGGCGGCGCGACCGGCC CGGGGCUCUUGGGCGGCCGCGU UUCCCCUCC
001,973	U6 snRNA	NCBI Accession Number: NR_004394	Name: RNA, U6 small nuclear 1, Symbol: RNU6-1	GTGCTCGCTTCGGCAGCACATATA CTAAAATTGGAACGATACAGAGAA GATTAGCATGGCCCCTGCGCAAGG ATGACACGCAAATTCGTGAAGCGTT CCATATTTT

^1^According to miRBase 22 (http://www.mirbase.org/)

The reverse transcription (RT) reaction was performed using a set of Custom RT Primer Pools and TaqMan MicroRNA Reverse Transcription Kit (both Applied Biosystems, Foster, CA, USA) according to the manufacturer’s protocol. In the next step, the preamplification reaction was performed using the TaqMan PreAmp Master Mix and Custom PreAmp Primer Pool (both Applied Biosystems) according to the manufacturer’s protocol.

The 7900HT Fast Real-Time PCR System and Custom TaqMan Array MircoRNA Card (both Applied Biosystems) were used to analyze the levels of selected miRNAs expression in the tested samples. U6 snRNA was used as the endogenous control. Relative miRNA expression was calculated using the 2^−ΔΔCT^ method [7] in the ExpressionSuite Software v1.03 implemented in the real-time PCR platform. The miRNA was assessed as “Detected” when the expression of a given miRNA was observed in the tested material. Cutoff for Ct numbers was 40 in ExpressionSuite Software.

Statistical analysis was performed using Statistica 13.5 (StatSoft Polska, Cracow, Poland) and Graph Pad Prism 7 (Graph Pad Software, San Diego, CA, USA), and a *p* value less than 0.05 was considered significant. Qualitative data were presented as numbers and percentages of the sample. The Chi-squared test (χ^2^) was used to compare the relationships between qualitative variables.

As the data were not normally distributed (Shapiro–Wilk test), Mann–Whitney and Kruskall-Wallis tests with subsequent post hoc analyses were used to compare the levels of miRNA expression between PACG, PEXG, and POAG. In addition, Spearman correlations were performed to examine the relationship between miRNA expression and selected clinical data. Data were expressed as mean with standard deviation (SD). Results were considered statistically significant when *p* ≤ 0.05.

Target annotation analysis and network visualization were performed using R environment (version 3.6.3, https://www.r-project.org) and appropriate packages according to corresponding reference manuals. Identification of miRNA/gene regulatory interactions was performed in silico between selected miRNAs and glaucoma-associated genes harvested from DisGeNET 7.0 database (https://www.disgenet.org/) [21–23], using multiMiR 1.10.0 package (https://bioconductor.org/packages/release/bioc/html/multiMiR.html, database version: 2.3.0, updated in 2020–04-15) [24]. Analysis included identification of both experimentally validated (miRecords, miRTarBase, and TarBase databases) and predicted (DIANA-microT-CDS, ElMMo, MicroCosm, miRanda, miRDB, PicTar, PITA, and TargetScan databases) miRNA/gene interactions.

Visualization of the regulatory network with obtained interactions was performed using Cytoscape v3.7.0 software (https://cytoscape.org/) [25].

Functional analysis of target genes was performed using Database for Annotation, Visualization and Integrated Discovery (DAVID) 6.8 tool (https://david.ncifcrf.gov/) [26, 27]. Default *Homo sapiens* genome was used as a background. Terms of Kyoto Encyclopedia of Genes and Genomes (KEGG), Reactome, and Gene Ontology (GO) categories were searched.

## Results

**Fig. 1:**
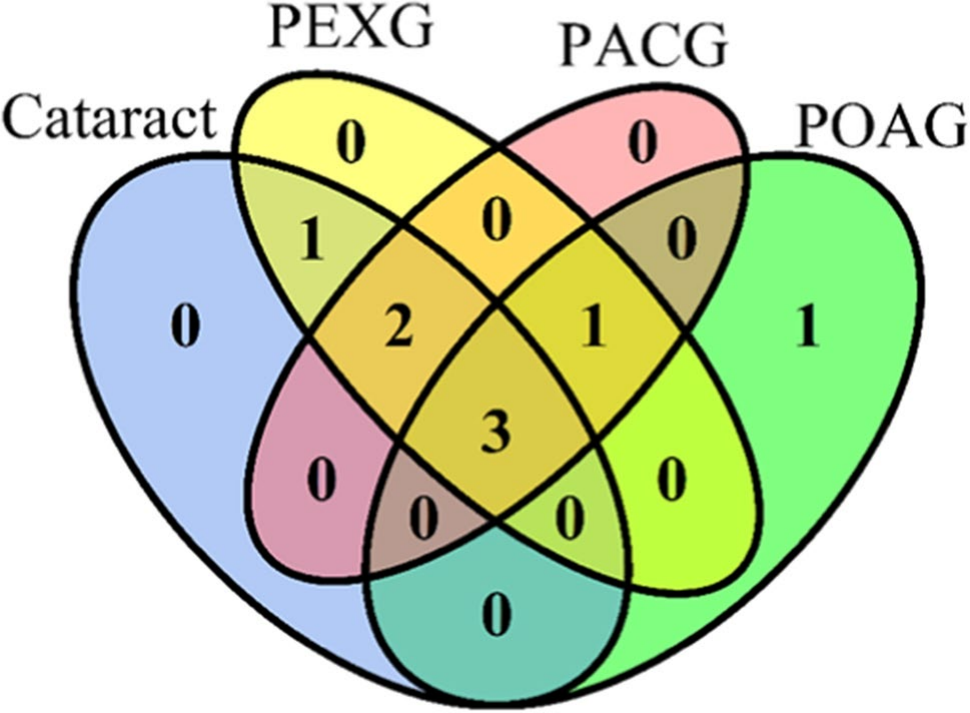
Venn diagram for miRNAs detected in at least 20% of samples within cataract, primary open angle glaucoma (POAG), pseudoexfoliation glaucoma (PEXG), and primary angle closure glaucoma (PACG) groups. Only in PAOG is hsa-miR-23a, common for only cataract and PEXG is hsa-miR-6722-3p, common for only PEXG, PACG, and POAG is hsa-miR-6515-3p, common for only cataract, PEXG and PACG are hsa-miR-184 and hsa-miR-187-5p, common for all groups are hsa-miR-1202, hsa-miR-1260b, and hsa-mir-4634. The plot was generated using VennDiagram 1.6.20 package in R

The ability to detect the expression of selected miRNAs in AH differed in studied glaucoma subgroups (Fig. 1, Table 3). Eight miRNAs were detected in at least 20% of samples in at least one of the studied groups. Three miRNAs (hsa-miR-1202, hsa-miR-1260b, hsa-mir-4634) were detected in at least 20% of samples in all studied groups (Fig. 1, Supplementary Table 1). The most frequently expressed miRNA in the studied panel was hsa-miR-1260b, which was detected in 19 POAG (100%), 13 PEXG (92%), and 7 PACG (77%) samples (Table 3, Supplementary Table 1).

**Table 3:** The frequency and the level of expression of the studied miRNAs, which expression enabled statistical analysis

miRNA ID	POAG		PEXG		PACG		Statistical analysis			POAG + PEXG + PACG	
	n	mean ± SD	n	mean ± SD	n	mean ± SD	p^1^	p^2^	p^3^	n	mean ± SD
hsa-miR-1202	4	− 0.44 ± 1.07	6	0.30 ± 3.10	2	− 0.47 ± 0.79	> 0.05	> 0.05	> 0.05	12	− 0.08 ± 2.21
hsa-miR-1260b	19	0.12 ± 0.79	13	− 0.16 ± 0.65	7	− 0.16 ± 0.51	> 0.05	> 0.05	> 0.05	39	− 0.06 ± 0.71
hsa-miR-184	3	0.65 ± 0.55	7	0.44 ± 0.80	2	− 0.03 ± 0.89	> 0.05	> 0.05	> 0.05	12	0.41 ± 0.73
hsa-miR-187-5p	3	2.45 ± 5.07	3	− 1.19 ± 0.11	2	− 0.30 ± 2.98	> 0.05	> 0.05	> 0.05	8	0.92 ± 3.40
hsa-mir-4634	12	− 0.09 ± 0.66	7	− 0.20 ± 0.50	4	− 0.16 ± 0.15	> 0.05	> 0.05	> 0.05	23	− 0.14 ± 0.54
hsa-miR-6515-3p	8	− 2.27 ± 0.90	3	0.21 ± 3.27	2	− 1.09 ± 0.52	> 0.05	> 0.05	> 0.05	13	− 1.52 ± 1.86
hsa-miR-6722-3p	3	0.93 ± 0.79	6	0.65 ± 2.25	1	− 0.71 ± N/A	> 0.05	> 0.05	> 0.05	10	0.60 ± 1.79

^1^Statistical significance of expression level between POAG, PEXG, PACG. ^2^Statistical significance of expression frequency. ^3^Statistical significance of expression level between control group and POAG + PEXG + PACG. *SD* standard deviation, *POAG* primary open angle glaucoma, *PEXG* pseudoexfoliation glaucoma, *PACG* primary angle closure glaucoma

**Fig. 2:**
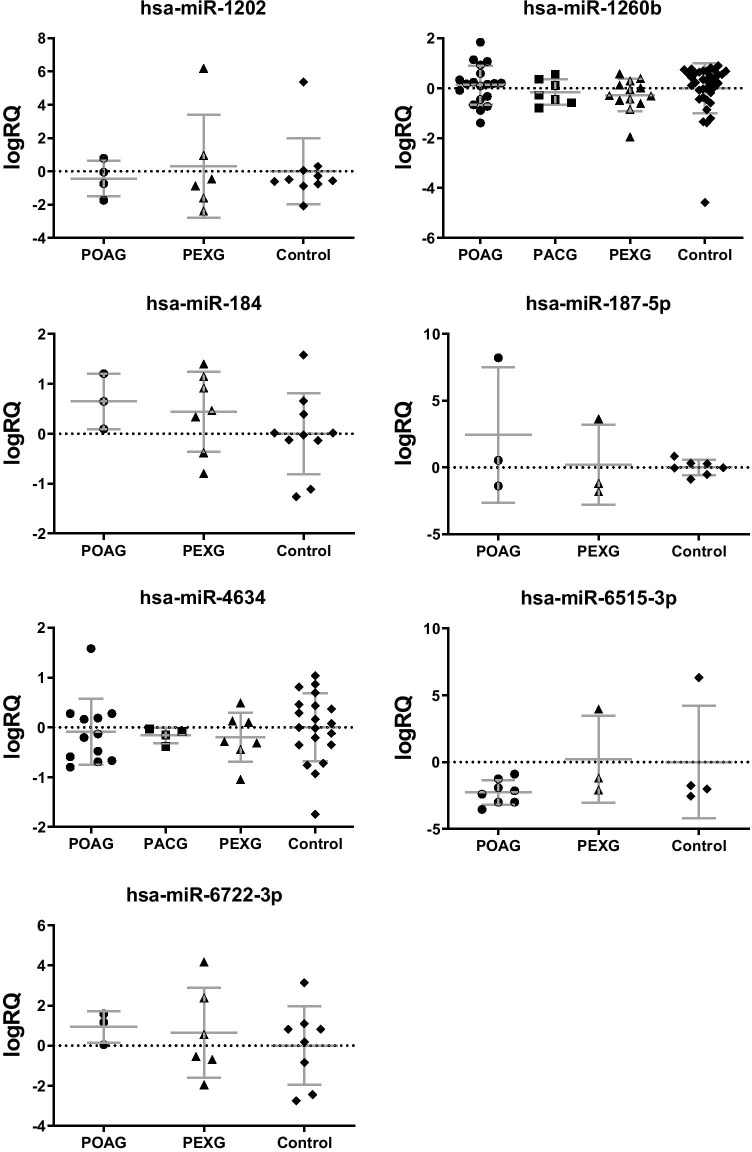
The expression levels of the most frequently detected miRNAs in the studied groups. Data are shown as mean ± standard deviation (SD). *POAG* primary open angle glaucoma, *PEXG* pseudoexfoliation glaucoma, *PACG* primary angle closure glaucoma

Seven out of eight the most frequently expressed miRNAs were detected in minimum 3 samples of two glaucoma subgroups and cataract group, enabling differential expression analysis (Table 3, Supplementary Table 2). We did not observe any significant differences in the frequency of expression and the level of expression for these miRNAs; however, there was a tendency for different expressions of hsa-miR-184, -1206b, and -6722-3p between various glaucoma types (Table 1, Fig. 2). Hsa-miR-184 (PEXG 50%, PACG 22%, POAG 16%, *p* = 0.0885) and hsa-miR-6722-3p (PEXG 43%, PACG 11%, POAG 16%, p = 0.1180) tended to express more frequently in PEXG patients (Table 3, Fig. 2).

We performed the correlation analysis between expression levels of seven of the most abundantly expressed miRNAs and the clinical status of the patients from the studied groups (Table 4). Interestingly, the expression of both hsa-miR-1260b (moderate correlation, *R* = 0.47) and hsa-miR-6515-3p (very strong correlation, R = 0.89) was statistically significantly correlated with age in POAG. The expression of hsa-miR-1260b was also correlated with the level of maximum IOP in the PACG group (very strong correlation, R = 0.89). This correlation was no significant in PEXG group, despite the similar level of IOP and hsa-miR-1260b expression. However, in the PEXG group, the expression levels of hsa-miR-1260b were correlated with the stage of glaucoma assessed as mean defect (MD) index in visual field (VF) examination (very strong correlation; *p* = 0.85). As MD is a negative value, it means that with a more advanced stage of glaucoma, the level of hsa-miR-1260b tended to decrease.

**Table 4:** Correlations of miRNA expression levels with age, maximum IOP and MD

miRNA	Group	Age	Maximum IOP	MD
hsa-miR-1260b	POAG	0.47*	0.16	− 0.01
	PACG	− 0.38	0.86*	0.32
	PEXG	− 0.19	− 0.21	0.85***
	Control	0.05	0.36	0.48*
hsa-miR-6515-3p	POAG	0.89**	0.16	− 0.07
	PACG	N/A	N/A	N/A
	PEXG	1.00	0.50	− 1.00
	Control	− 0.20	0.47	− 0.95
hsa-miR-4634	POAG	0.40	0.07	− 0.38
	PACG	0.40	0.40	0.40
	PEXG	− 0.36	0.38	0.29
	Control	0.26	0.58*	0.01
hsa-miR-1202	POAG	− 0.63	− 0.40	0.40
	PACG	N/A	N/A	N/A
	PEXG	− 0.26	0.23	0.20
	Control	0.36	− 0.84*	0.87
hsa-miR-184	POAG	0.50	0.50	1.00
	PACG	N/A	N/A	N/A
	PEXG	− 0.33	0.22	0.57
	Control	− 0.36	0.37	− 0.27
hsa-miR-6722-3p	POAG	1.00	0.50	− 0.50
	PACG	N/A	N/A	N/A
	PEXG	0.61	− 0.23	− 0.43
	Control	− 0.95*	0.63	− 0.40
hsa-miR-187	POAG	− 0.50	− 1.00	− 0.50
	PACG	N/A	N/A	N/A
	PEXG	− 0.50	0.00	− 0.50
	Control	0.20	0.63	− 0.50

^*^p ≤ 0.05; ** *p* ≤ 0.01; *** *p* ≤ 0.001; *IOP* intraocular pressure, *MD* mean defect, *N/A* not calculated due to low frequency of detection, *POAG* primary open angle glaucoma, *PEXG* pseudoexfoliation glaucoma, *PACG* primary angle closure glaucoma

**Fig. 3:**
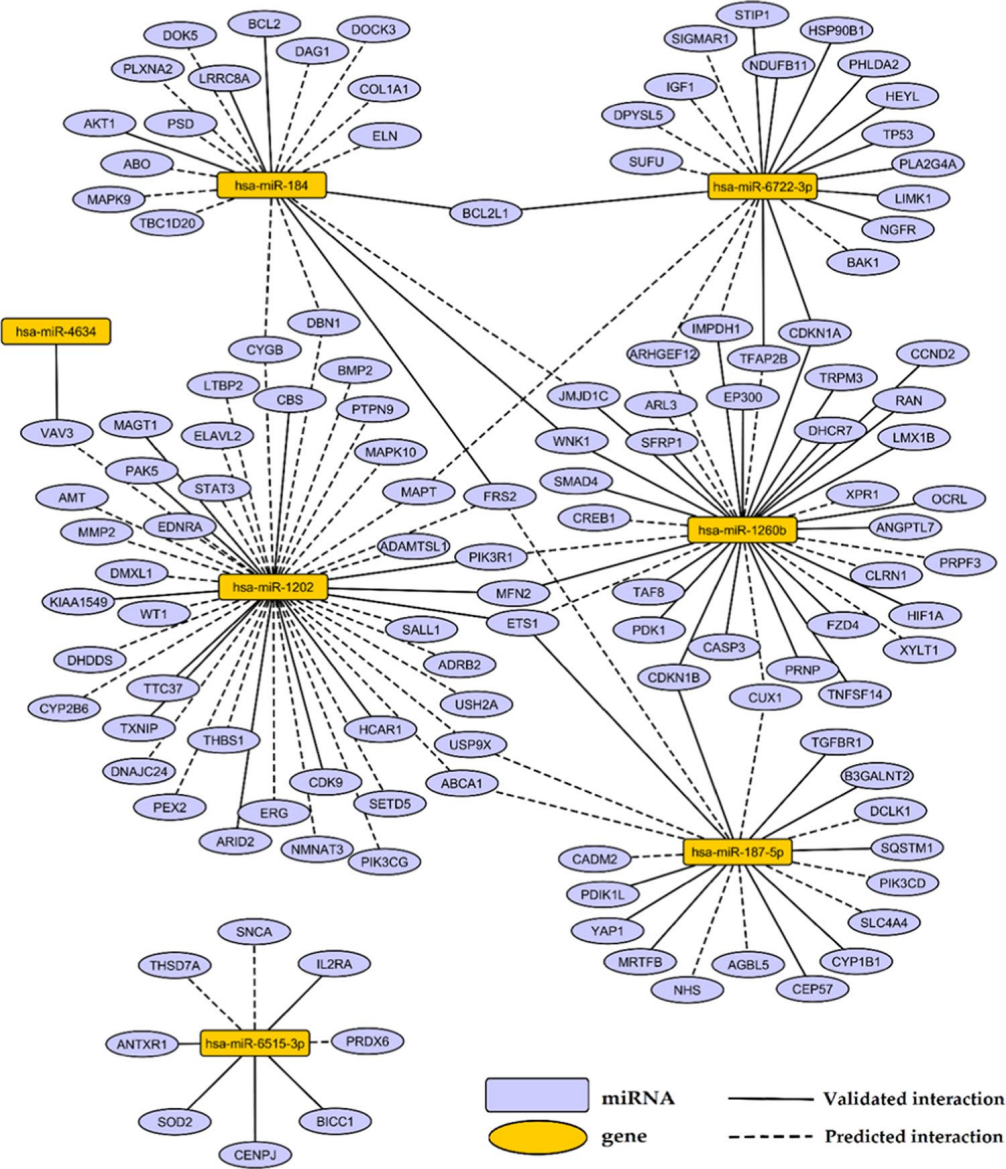
Regulatory network presenting interactions between seven most frequently expressed miRNAs in glaucoma and genes obtained from DisGeNET 7.0 database as associated with glaucoma. Interactions were found in silico using multiMiR 1.10.0 package in R

To recognize a regulatory function of analyzed miRNAs in glaucoma pathology, we performed in silico target annotation analysis between seven the most frequently expressed miRNAs found in AH of patients with glaucoma (presented in Table 1) and 770 glaucoma-associated genes received from DisGeNET 7.0 database (Concept Unique Identifier “C0017601” was queried). Target annotation analysis requires mature miRNA IDs as input; therefore in the case of hsa-mir-4634 stem-loop miRNA, we used its-derived mature miRNA ID: hsa-miR-4634. Target annotation analysis revealed 69 validated miRNA:gene pairs (Supplementary Table 3) as well as 86 top 10% predicted miRNA:gene pairs obtained with the highest probability (Supplementary Table 4). Identified interactions were visualized on the regulatory network (Fig. 3).

**Fig. 4:**
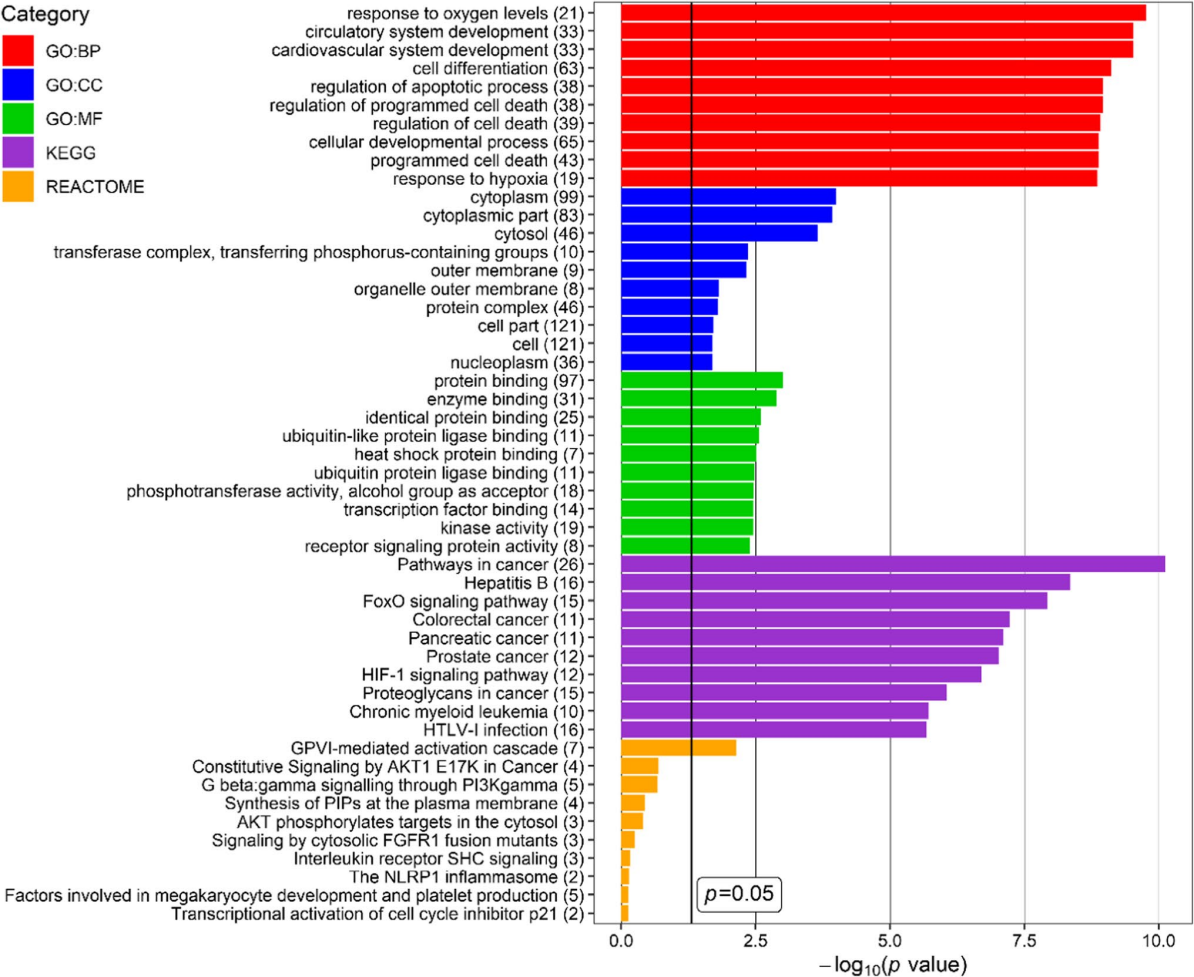
Top ten the most enriched terms of GO (Gene Ontology) Biological Processing (GO:BP), GO Cellular Compartment (GO:CC), GO Molecular Function (GO:MF), KEGG (Kyoto Encyclopedia of Genes and Genomes) and REACTOME categories, revealed for glaucoma-associated genes targeted by miRNAs found in the current study as the most frequently expressed in aqueous humour (AH) of patients with glaucoma. *P* value – EASE score for enrichment adjusted by Benjamini correction for multiple hypothesis testing. The number in brackets following name of terms indicates number of associated genes. The plot was generated using ggplot2 3.3.0 package in R

To indicate biological processes in which miRNA-regulated genes are involved, functional analysis was performed for 124 networked genes using DAVID website tool. Figure 4 presents top ten the most enriched terms of GO (Gene Ontology) Biological Processing, GO Cellular Compartment, GO Molecular Function, KEGG (Kyoto Encyclopedia of Genes and Genomes), and Reactome categories.

Genes found as targets of the most frequently expressed miRNAs in glaucoma were associated with the cardiovascular system development, response to hypoxia, apoptosis, cytoplasm compartment, enzymatic activity, cancer, FoxO, PI3K-AKT and HIF-1 signalling pathways, viral infections, blood coagulation cascade, and regulation of inflammation.

## Discussion

Extracellular miRNAs, both secreted by intraocular cells and derived from blood plasma, play essential role in ocular development and retinal homeostasis [28]. The level of miRNAs in AH was shown to be influenced by damages in the anterior and posterior ocular segment [4]. Moreover, trabecular meshwork cell contractility and extracellular matrix turnover are influenced by distinct miRNAs [29–31] that may therefore be relevant in glaucoma. In the current study, expression of 18 selected miRNA was profiled in AH of patients with different types of glaucoma (POAG, PACG and PEXG) as well as in cataract group as control to check whether the expression patterns of miRNA may reflect different mechanisms causing IOP elevation.

In our study, seven miRNAs were revealed as the most abundant in AH of glaucoma patients (Table 1, Fig. 2). However, expression of these miRNAs did not significantly differentiate glaucoma and cataract patients as well as glaucoma subgroups. Our findings are consistent with previous studies, where any of these seven miRNAs were not reported as differentially expressed with statistical significance [4, 32, 33].

In our research, the most frequently detected miRNAs in AH of glaucoma patients were hsa-miR-4634 and hsa-miR-1260b (Table 1, Fig. 2). Hsa-miR-4634 is a validated regulator of *VAV3* (Fig. 3), whose deficiency in mice was associated with an ocular phenotype similar to glaucoma, including elevated IOP, selective loss of retinal ganglion cells and optic nerve head cupping [34]. Hsa-miR-1260b tended to be expressed more frequently in POAG patients. This miRNA was previously shown to target genes regulating proliferation and differentiation of neuronal cells, e.g., *LMX1B* [35], *SMAD4* [36], *WNK1* [37], and *CREB1* [38]. Genetic variants and mutations in *LMX1B* were previously associated with susceptibility of glaucoma [39–42]. It suggests a possible role of hsa-miR-1260b in glaucoma by controlling cellular differentiation and proliferation; however the mechanism should be further elucidated.

There are data showing the influence of some miRNA variants on glaucoma endophenotype. Ghanbari et al. [43] found that variants in the hsa-miR-612 precursor and in the hsa-miR-4707 seed region were significantly associated with vertical cup-to-disc ratio and cup area. In this study a strong positive correlation between hsa-miR-1260b and MD was observed, which shows that the level of hsa-miR-1260b expression decreased in more advanced PEXG cases. It suggests that hsa-miR-1260b may play a protective role in the course of glaucomatous neuropathy. On the other hand, *CREB*, one of the genes targeted by hsa-miR-1260b, has a neuroprotective effect against hydrogen peroxide-induced retinal ganglion cell death via two downstream cell survival genes, *BDNF* and *BCL2* [44]. Upregulation of *CREB* and *BCL2* enhances cell survival and reduces apoptosis after optic nerve crush [45] as well as activate MEK/ERK/CREB pathway as a protective mechanism of silibinin against blue light damage of retinal ganglion cells [46]. The effect of interaction between hsa-miR-1260b and *CREB* should be investigated in further studies.

Hsa-miR-1260b is also an essential regulator of vascular smooth muscle cells proliferation in response to hypoxia [47]. In all areas of the trabecular meshwork and especially in the area external to Schlemm’s canal, there are cells that have cytoplasm rich in actin filaments and have characteristics common to smooth muscle cells [48] and thus may play a role in AH outflow modification. In our study, we observed significant positive correlation between the expression of hsa-miR-1260b and maximum IOP in PACG patients (Table 4), which suggests a probable relation of hsa-miR-1260b to mechanisms of IOP increase. However, it may be a part of some PACG-specific pathomechanisms because the IOP level did not differ in patients with PACG and POAG, and in the case of the latter, the correlation between the level of hsa-miR-1260b expression and IOP was not significant.

Additionally, in our research, hsa-miR-1260b was found to be a validated target of *SMAD4* and *SFRP1* (Fig. 3), which are involved in the outflow regulatory mechanisms in eye anterior chamber. These two genes participate in the inhibitory crosstalk between TGFβ/Smad and Wnt signaling pathways, previously described in human glaucomatous trabecular meshwork cells [49]. Activation of TGFβ/Smad signaling and upregulation of *SMAD4* were reported in oxidative stress treated human trabecular meshwork cells and was associated with increased production of extracellular matrix [50]. Wnt signaling and its key mediator β-catenin were reported to be inhibited in elevated IOP by upregulation of *SFRP1* [49]. Activation of Wnt pathway in glaucoma alleviated detrimental effects of overactivated TGFβ/Smad signaling, since Wnt signaling pathway facilitates AH outflow and decreases IOP [51, 52]. Therefore, the protective effect of hsa-miR-1260b could result from inhibition of *SMAD4* and *SFRP1*.

Another miRNA detected in all studied groups is hsa-miR-184, which is one of the most abundantly expressed miRNAs in normal human ciliary body, cornea, and trabecular meshwork [53]. Similarly, hsa-miR-184 is also very abundant in AH, with a level much higher than in plasma [54]. This miRNA is also highly expressed in both transparent and cataract lens samples [55]. Prior studies reported high expression of this miRNA in AH of patients with POAG and cataract, however without statistical significant difference [4], which is in consistence with our results (Table 1). We additionally observed the tendency of hsa-miR-184 to be expressed more frequently in AH samples of patients with pseudoexfoliation syndrome, which is the risk factor not only for glaucoma but also for cataract, indicating a potential role of this miRNA in both conditions. Previous studies reported four different point mutations in hsa-miR-184, which were linked with lens/corneal dystrophy and blindness [56–59]; however, function of hsa-miR-184 under homeostasis and its relation to eye pathology remain to be investigated [8].

Apoptosis and dendritic detraction of retinal ganglion cells are main processes contributing to each type of glaucoma [60]. Target annotation analysis performed in silico in our study showed that hsa-miR-184 targets genes controlling apoptosis, including *BCL2*, *BCL2L1*, *MAPK9*, and *AKT1* (Fig. 2). Possible repression of these anti-apoptotic genes mediated by interactions with hsa-miR-184 may contribute to potentiate apoptosis potentially aggravating glaucoma outcome; however, this hypothesis requires further verification in experimental studies. Other miRNAs abundantly expressed in AH of glaucoma patients were also showed in silico in our study as regulators of genes related to apoptosis, e.g., *STAT3*, *BMP2* (targets for hsa-miR-1202), *CASP3*, *PDKI* (targets for hsa-miR-1260b), *BAK1,* and *TP53* (targets for hsa-miR-6722-3p) (Fig. 2). These findings indicate that miRNAs are involved in regulation of cell apoptosis and survival during glaucoma course and further studies are needed to elucidate this mechanism.

The proper AH flow is maintained by trabecular meshwork cells, which exhibit high susceptibility to injury induced by oxidative free radicals. Dysfunctions of these cells caused by excessive oxygen species may contribute to increase in IOP and glaucoma [10, 11]. There is a piece of evidence that oxidative stress, together with mitochondrial impairment and pathogenic events, contributes to a complex network of mechanisms leading to glaucoma [12, 13, 61]. Interestingly, target annotation analysis revealed validated interactions of hsa-miR-6515-3p with eight genes (Fig. 2) and two out of them, *PRDX6* and *SOD2*, were previously associated with response to oxidative stress, which is a major cause of glaucoma pathogenesis [62]. *PRDX6* was previously shown to delay senescence and limit reactive oxygen species accumulation in human trabecular meshwork cells, and its downregulation was observed in aged and glaucomatous trabecular meshwork cells [63]. *SOD2* encodes the manganese-dependent superoxide dismutase that acts as a mitochondrial antioxidant enzyme [64], and higher level of SOD enzyme was evidenced in AH of POAG patients in comparison to cataract subjects [65]. Targeting of *PRDX6* and *SOD2* by hsa-miR-6515-3p could be a possible mechanism promoting cellular senescence and impairing antioxidant ability, thus also contributing to glaucoma. Additionally, many polymorphisms identified in *SOD2* and *PRDX6* genes [66–68] may potentially influenced interactions with miRNAs affecting susceptibility to glaucoma.

Wang et al. [9] demonstrated that miRNAs could be oxidised in response to oxidative stress. Upon oxidative modification, hsa-miR-184 misrecognizes mRNA for Bcl-xL and Bcl-w, which are not its native targets, resulting in initiation of cellular apoptosis. Moreover, Gartaganis et al. [14] found that in PEX syndrome, AH samples showed a decrease in glutathione concentration and an increase in glutathione disulfide level, which suggests that oxidation stress plays a role in the PEX pathogenesis and progression. Reduced levels of selenium in AH, conjunctival specimens, and serum of PEX patients also support the hypothesis that the impairment of the antioxidant defense system participates in PEX pathogenesis [69]. Thus, a higher frequency of hsa-miR-184 expression in the PEXG group observed in this study may support previous results.

In glaucoma pathogenesis, blood flow reduction and ischemia are postulated as possible causative mechanisms of optic nerve neuropathy. Reperfusion injury, one of proposed mechanisms, refers to damage to nerve tissue caused when blood supply improves to the tissue after a period of ischemia. The absence of oxygen and nutrients from tissue creates a condition in which the restoration of circulation results in inflammation and oxidative damage rather than restoration of normal function [70]. One of the most enriched functional terms of genes targeted by selected seven the most frequently expressed miRNAs in AH of patients with glaucoma was “response to oxygen levels” and “response to hypoxia” (Fig. 3). Our in silico target annotation analysis showed that hsa-miR-1260b, hsa-miR-1202, and hsa-miR-187-5p target *ETS1* (Fig. 2), which is a transcription factor undergoing upregulation under hypoxia condition and participate in induction of hypoxia-inducible genes expression [71]. Besides *ETS1*, hsa-miR-1260b targets other hypoxia-associated genes, like *HIF1* and *PDK1* [72] (Fig. 2). The potential role of this miRNA in response to hypoxia in glaucoma neuropathy should be further elucidated.

Apart from elevated IOP, the major risk factor for POAG is older age [73]. In this study, we observed a correlation between age and expression of both hsa-miR-6515-3p and hsa-miR-1260b in POAG patients, which may show that these miRNAs regulate aging-related processes contributing to glaucoma pathogenesis. An association between glaucoma and neurodegenerative diseases of the central nervous system, such as Alzheimer’s disease (AD), age-related dementia with progressive deterioration of memory and cognition, has also been reported [74]. In our study, hsa-miR-184 and hsa-miR-6722-3p tended to be expressed more frequently in PEXG patients when compared with POAG and PACG groups (Fig. 2) and with the mean level of expression slightly lower than in POAG (difference not significant). Kumar et al. [15] found that miR-6722 was downregulated in serum of patients with AD. Decrease in expression of MiR-184 was found in hippocampus of patients with late-onset AD [75]. The major pathological hallmarks of AD include accumulation of large extracellular β‐amyloid plaques and intracellular fibrillary tangles of abnormally phosphorylated tau protein. The deposition of amyloid‐like material in PEX syndrome shares some features with the findings in AD [16]. Amyloid‐β peptide has also been demonstrated in AH of PEX patients [17]. In keeping with these observations, a possible relationship between PEX and AD has been suggested, which is consistent with our results. Additionally, Inoue et al. [18] found that PEXG and POAG patients manifested elevated levels of some AD biomarkers (apolipoprotein E, transthyretin) in AH, and this level was related to the severity of glaucoma.

This study has some limitations. First, the control group constitutes of patients with senile cataract without glaucoma. The impact of cataract in glaucoma subgroups on miRNA expression should be taken into consideration, as part of the dysregulated miRNAs found in previous studies may be related to both cataract and glaucoma [4]. Moreover, the number of evaluated samples was not equal for all studied groups. It partially reflects the incidence rate of glaucoma in our population and is also connected with the technical difficulty of obtaining the AH samples from the patients with shallow anterior chamber (PACG). Finally, the study evaluates only the group of miRNAs selected after the careful reviewing of the publications available at the time of the study design [19, 20] concerning the subject at the time of study design. The authors decided to select the miRNA most frequently expressed, according to these publications, in the aqueous humor. Although this was the only possible strategy, the authors are aware of the possible bias caused by tightening of the studied panel.

To sum up, in this study we were able to show the expression of selected miRNAs in AH of glaucoma patients. The level of expression was similar for various glaucoma types, but the frequency of expression tended to differ. Additionally, some correlations between clinical features and the studied miRNAs were found, which confirms the possibility that miRNAs influence glaucoma endophenotype. Potential functions of genes targeted by the most frequently detected miRNAs were presented, indicating the important role of miRNAs in glaucoma pathology.

## Supplementary Information

Below is the link to the electronic supplementary material.

Supplementary file1 (DOCX 810 KB)

## Data Availability

All data and materials support our published claims and comply with field standards.
