# Supplementary material for: MicroRNAs in the aqueous humor of patients with different types of glaucoma
Source: Graefes Arch Clin Exp Ophthalmol. 2021 Apr 30;259(8):2337–49. doi: 10.1007/s00417-021-05214-z (PMC8352835; doi:10.1007/s00417-021-05214-z)
Supplement: Supplementary file 1 — Supplementary file1 (DOCX 810 KB) [file 417_2021_5214_MOESM1_ESM.docx]

**SUPPLEMENTARY MATERIAL**

**Supplementary Tables:**

**Table S1.** The number and fraction of samples in which studied miRNAs were detected.

| miRNA  (miRBase ID) | Studied groups: | | | |
| --- | --- | --- | --- | --- |
|  | **Cataract**  **n = 36** | **POAG**  **n = 19** | **PEXG**  **n = 14** | **PACG**  **n = 9** |
| hsa-let-7b-3p | 3 (8.3%) | 1 (5.3%) | 2 (14.3%) | 1 (11.1%) |
| hsa-miR-1202 | 10 (27.8%) | 4 (21.1%) | 6 (42,9%) | 2 (22.2%) |
| hsa-miR-125b-5p | 5 (13.9%) | 1 (5.3%) | 1 (7.1%) | 1 (11.1%) |
| hsa-miR-1260b | 36 (100%) | 19 (100%) | 13 (92.9%) | 7 (77.8%) |
| hsa-miR-184 | 10 (27.8%) | 3 (15.8%) | 7 (50%) | 2 (22.2%) |
| hsa-miR-187-5p | 8 (22.2%) | 3 (15.8%) | 3 (21.4%) | 2 (22.2%) |
| hsa-miR-202-5p | 4 (11.1%) | 0 (0%) | 0 (0%) | 0 (0%) |
| hsa-miR-23a-3p | 1 (2.8%) | 4 (21.1%) | 0 (0%) | 1 (11.1%) |
| hsa-miR-3197 | 3 (8.3%) | 2 (10.5%) | 0 (0%) | 0 (0%) |
| hsa-miR-3663-3p | 1 (2.8%) | 1 (5.3%) | 0 (0%) | 0 (0%) |
| hsa-miR-3940-5p | 4 (11.1%) | 2 (10.5%) | 0 (0%) | 0 (0%) |
| hsa-miR-4433a-3p | 3 (8.3%) | 1 (5.3%) | 0 (0%) | 1 (11.1%) |
| hsa-miR-4467 | 2 (5.6%) | 2 (10.5%) | 1 (7.1%) | 1 (11.1%) |
| hsa-miR-4484 | 2 (5.6%) | 0 (0%) | 0 (0%) | 0 (0%) |
| hsa-mir-4634 | 20 (55.6%) | 12 (63.2%) | 7 (50%) | 4 (44.4%) |
| hsa-miR-4725-3p | 4 (11.1%) | 1 (5.3%) | 1 (7.1%) | 1 (11.1%) |
| hsa-miR-4749-5p | 0 (0%) | 1 (5.3%) | 0 (0%) | 1 (11.1%) |
| hsa-miR-5001-5p | 1 (2.8%) | 1 (5.3%) | 0 (0%) | 0 (0%) |
| hsa-miR-6132 | 0 (0%) | 0 (0%) | 0 (0%) | 0 (0%) |
| hsa-miR-6515-3p | 4 (11.1%) | 8 (42.1%) | 3 (21.4%) | 2 (22.2%) |
| hsa-miR-6717-5p | 3 (8.3%) | 1 (5.3%) | 0 (0%) | 0 (0%) |
| hsa-miR-6722-3p | 8 (22.2%) | 3 (15.8%) | 6 (42.9%) | 1 (11.1%) |

POAG - primary open angle glaucoma, PEXG - pseudoexfoliation glaucoma, PACG - primary angle closure glaucoma

**Table S2.** The frequency and the expression levels of the studied miRNAs in glaucoma subgroups in relative to cataract group as control.

| **miRNA**  **(miRBase ID)** | **Glaucoma subgroups:** | | | | | |
| --- | --- | --- | --- | --- | --- | --- |
|  | **POAG** | | **PEXG** | | **PACG** | |
|  | **n** | **mean±SD** | **n** | **mean±SD** | **n** | **mean±SD** |
| hsa-let-7b-3p | 1 | 0,17± N/A | 2 | 1,55±0,31 | 1 | -4,74±N/A |
| hsa-miR-1202 | 4 | -0.44±1.07 | 6 | 0.30±3.10 | 2 | -0.47±0.79 |
| hsa-miR-125b-5p | 1 | 2,74± N/A | 1 | 1,60± N/A | 1 | 8,26± N/A |
| hsa-miR-1260b | 19 | 0.12±0.79 | 13 | -0.16±0.65 | 7 | -0.16±0.51 |
| hsa-miR-184 | 3 | 0.65±0.55 | 7 | 0.44±0.80 | 2 | -0.03±0.89 |
| hsa-miR-187-5p | 3 | 2.45±5.07 | 3 | -1.19±0.11 | 2 | -0.30±2.98 |
| hsa-miR-202-5p | 0 | N/A±N/A | 0 | N/A±N/A | 0 | N/A±N/A |
| hsa-miR-23a-3p | 4 | 3,37±1,08 | 0 | N/A±N/A | 1 | -3,00±N/A |
| hsa-miR-3197 | 2 | 0,32±6,79 | 0 | N/A±N/A | 0 | N/A±N/A |
| hsa-miR-3663-3p | 1 | 2,66± N/A | 0 | N/A±N/A | 0 | N/A±N/A |
| hsa-miR-3940-5p | 2 | -0,70±0,08 | 0 | N/A±N/A | 0 | N/A±N/A |
| hsa-miR-4433a-3p | 1 | -2,05±N/A | 0 | N/A±N/A | 1 | 0,21± N/A |
| hsa-miR-4467 | 2 | 0,80±0,03 | 1 | 0,25± N/A | 1 | 1,35± N/A |
| hsa-miR-4484 | 0 | N/A±N/A | 0 | N/A±N/A | 0 | N/A±N/A |
| hsa-mir-4634 | 12 | -0.09±0.66 | 7 | -0.20±0.50 | 4 | -0.16±0.15 |
| hsa-miR-4725-3p | 1 | -3,36±N/A | 1 | 1,91± N/A | 1 | -2,52±N/A |
| hsa-miR-4749-5p | 1 | N/A±N/A | 0 | N/A±N/A | 1 | N/A±N/A |
| hsa-miR-5001-5p | 1 | -6,96±N/A | 0 | N/A±N/A | 0 | N/A±N/A |
| hsa-miR-6132 | 0 | N/A±N/A | 0 | N/A±N/A | 0 | N/A±N/A |
| hsa-miR-6515-3p | 8 | -2.27±0.90 | 3 | 0.21±3.27 | 2 | -1.09±0.52 |
| hsa-miR-6717-5p | 1 | -3,35±N/A | 0 | N/A±N/A | 0 | N/A±N/A |
| hsa-miR-6722-3p | 3 | 0.93±0.79 | 6 | 0.65±2.25 | 1 | -0.71±N/A |

N/A – not applicable, n - the number of samples in which miRNA was detected, POAG - primary open angle glaucoma, PEXG - pseudoexfoliation glaucoma, PACG - primary angle closure glaucoma

**Table S3.** Experimentally validated miRNA:gene pairs found *in silico* among 7 the most frequently detected miRNAs in AH of patients with glaucoma and 770 genes associated with glaucoma.

| **No.** | **miRNA miRBase ID** | **Gene symbol** | **Database** | **Validation method** |
| --- | --- | --- | --- | --- |
|  | hsa-miR-1202 | *ARID2* | tarbase | Degradome sequencing |
|  | hsa-miR-1202 | *CBS* | mirtarbase | PAR-CLIP |
|  | hsa-miR-1202 | *CDK9* | tarbase | Degradome sequencing |
|  | hsa-miR-1202 | *ETS1* | mirtarbase | HITS-CLIP |
|  | hsa-miR-1202 | *HCAR1* | tarbase | Degradome sequencing |
|  | hsa-miR-1202 | *KIAA1549* | tarbase | Degradome sequencing |
|  | hsa-miR-1202 | *MAGT1* | mirtarbase | HITS-CLIP |
|  | hsa-miR-1202 | *MFN2* | tarbase | Degradome sequencing |
|  | hsa-miR-1202 | *PAK5* | mirtarbase | HITS-CLIP |
|  | hsa-miR-1202 | *PIK3R1* | mirtarbase | PAR-CLIP |
|  | hsa-miR-1202 | *TTC37* | tarbase | Degradome sequencing |
|  | hsa-miR-1202 | *TXNIP* | mirtarbase | PAR-CLIP |
|  | hsa-miR-1260b | *ANGPTL7* | mirtarbase | HITS-CLIP |
|  | hsa-miR-1260b | *CASP3* | tarbase | Degradome sequencing |
|  | hsa-miR-1260b | *CCND2* | tarbase | Degradome sequencing |
|  | hsa-miR-1260b | *CDKN1A* | mirtarbase | CLASH |
|  | hsa-miR-1260b | *CDKN1B* | mirtarbase | CLASH |
|  | hsa-miR-1260b | *DHCR7* | mirtarbase | CLASH |
|  | hsa-miR-1260b | *EP300* | tarbase | Degradome sequencing |
|  | hsa-miR-1260b | *FZD4* | tarbase | Degradome sequencing |
|  | hsa-miR-1260b | *HIF1A* | tarbase | Degradome sequencing |
|  | hsa-miR-1260b | *IMPDH1* | tarbase | Degradome sequencing |
|  | hsa-miR-1260b | *JMJD1C* | tarbase | Degradome sequencing |
|  | hsa-miR-1260b | *LMX1B* | mirtarbase | HITS-CLIP |
|  | hsa-miR-1260b | *MFN2* | mirtarbase | CLASH |
|  | hsa-miR-1260b | *OCRL* | tarbase | Degradome sequencing |
|  | hsa-miR-1260b | *PDK1* | tarbase | Degradome sequencing |
|  | hsa-miR-1260b | *PRNP* | mirtarbase | PAR-CLIP |
|  | hsa-miR-1260b | *RAN* | mirtarbase | CLASH |
|  | hsa-miR-1260b | *SFRP1* | mirtarbase, tarbase | Luciferase reporter assay, qRT-PCR, Western blot, degradome sequencing |
|  | hsa-miR-1260b | *SMAD4* | mirtarbase, tarbase | Luciferase reporter assay, qRT-PCR, Western blot, degradome sequencing |
|  | hsa-miR-1260b | *TAF8* | mirtarbase | HITS-CLIP |
|  | hsa-miR-1260b | *TNFSF14* | mirtarbase | PAR-CLIP |
|  | hsa-miR-1260b | *TRPM3* | mirtarbase | PAR-CLIP |
|  | hsa-miR-1260b | *WNK1* | tarbase | Degradome sequencing |
|  | hsa-miR-184 | *AKT1* | mirtarbase | Luciferase reporter assay |
|  | hsa-miR-184 | *BCL2* | mirtarbase | Luciferase reporter assay |
|  | hsa-miR-184 | *BCL2L1* | mirtarbase | PAR-CLIP |
|  | hsa-miR-184 | *FRS2* | tarbase | Degradome sequencing |
|  | hsa-miR-184 | *LRRC8A* | mirtarbase | PAR-CLIP |
|  | hsa-miR-184 | *WNK1* | tarbase | Degradome sequencing |
|  | hsa-miR-187-5p | *B3GALNT2* | mirtarbase | PAR-CLIP |
|  | hsa-miR-187-5p | *CDKN1B* | mirtarbase | PAR-CLIP |
|  | hsa-miR-187-5p | *CEP57* | tarbase | Degradome sequencing |
|  | hsa-miR-187-5p | *CYP1B1* | mirtarbase | Luciferase reporter assay, qRT-PCR, Western blot |
|  | hsa-miR-187-5p | *ETS1* | tarbase | Degradome sequencing |
|  | hsa-miR-187-5p | *MRTFB* | tarbase | Degradome sequencing |
|  | hsa-miR-187-5p | *PDIK1L* | mirtarbase | PAR-CLIP |
|  | hsa-miR-187-5p | *SQSTM1* | tarbase | Degradome sequencing |
|  | hsa-miR-187-5p | *TGFBR1* | tarbase | Degradome sequencing |
|  | hsa-miR-187-5p | *YAP1* | tarbase | Degradome sequencing |
|  | hsa-miR-4634 | *VAV3* | mirtarbase | HITS-CLIP, PAR-CLIP |
|  | hsa-miR-6515-3p | *ANTXR1* | mirtarbase | HITS-CLIP |
|  | hsa-miR-6515-3p | *BICC1* | mirtarbase | HITS-CLIP |
|  | hsa-miR-6515-3p | *CENPJ* | mirtarbase | HITS-CLIP |
|  | hsa-miR-6515-3p | *IL2RA* | mirtarbase | HITS-CLIP |
|  | hsa-miR-6515-3p | *SOD2* | mirtarbase | PAR-CLIP |
|  | hsa-miR-6722-3p | *BCL2L1* | mirtarbase | PAR-CLIP |
|  | hsa-miR-6722-3p | *CDKN1A* | mirtarbase | PAR-CLIP |
|  | hsa-miR-6722-3p | *HEYL* | mirtarbase | PAR-CLIP |
|  | hsa-miR-6722-3p | *HSP90B1* | mirtarbase | PAR-CLIP |
|  | hsa-miR-6722-3p | *LIMK1* | mirtarbase | PAR-CLIP |
|  | hsa-miR-6722-3p | *NDUFB11* | mirtarbase | PAR-CLIP |
|  | hsa-miR-6722-3p | *NGFR* | mirtarbase | PAR-CLIP |
|  | hsa-miR-6722-3p | *PHLDA2* | mirtarbase | HITS-CLIP |
|  | hsa-miR-6722-3p | *PLA2G4A* | mirtarbase | HITS-CLIP |
|  | hsa-miR-6722-3p | *STIP1* | mirtarbase | PAR-CLIP |
|  | hsa-miR-6722-3p | *TFAP2B* | mirtarbase | PAR-CLIP |
|  | hsa-miR-6722-3p | *TP53* | mirtarbase | PAR-CLIP |

CLASH – Cross-linking, Ligation, and Sequencing of Hybrids, HITS-CLIP – High-Throughput Sequencing of RNA isolated by Cross-Linking Immunoprecipitation, PAR-CLIP – Photoactivatable Ribonucleoside-Enhanced Crosslinking and Immunoprecipitation, qRT-PCR – Quantitative Reverse-Transcriptase Polymerase Chain Reaction,

**Table S4.** Top 10% predicted miRNA:gene pairs obtained in silico among 7 the most frequently detected miRNAs in AH of patients with glaucoma and 770 genes associated with glaucoma.

| **No.** | **miRNA miRBase ID** | **Gene symbol** | **Database** | **Database-specific probability value** |
| --- | --- | --- | --- | --- |
|  | hsa-miR-1202 | *ABCA1* | diana_microt | 0.821 |
|  | hsa-miR-1202 | *ABCA1* | mirdb | 95.6 |
|  | hsa-miR-1202 | *ABCA1* | pita | -11.77 |
|  | hsa-miR-1202 | *ADAMTSL1* | diana_microt | 0.805 |
|  | hsa-miR-1202 | *ADRB2* | pita | -9.43 |
|  | hsa-miR-1202 | *AMT* | diana_microt | 0.807 |
|  | hsa-miR-1202 | *ARID2* | pita | -10.71 |
|  | hsa-miR-1202 | *BMP2* | pita | -12.8 |
|  | hsa-miR-1202 | *CYGB* | pita | -16.96 |
|  | hsa-miR-1202 | *CYP2B6* | pita | -10.32 |
|  | hsa-miR-1202 | *DBN1* | pita | -10.28 |
|  | hsa-miR-1202 | *DHDDS* | pita | -12.09 |
|  | hsa-miR-1202 | *DHDDS* | pita | -17.54 |
|  | hsa-miR-1202 | *DMXL1* | pita | -10.95 |
|  | hsa-miR-1202 | *DNAJC24* | pita | -10.22 |
|  | hsa-miR-1202 | *EDNRA* | pita | -9.46 |
|  | hsa-miR-1202 | *ELAVL2* | pita | -9.45 |
|  | hsa-miR-1202 | *ERG* | diana_microt | 0.882 |
|  | hsa-miR-1202 | *ETS1* | diana_microt | 0.987 |
|  | hsa-miR-1202 | *ETS1* | pita | -9.95 |
|  | hsa-miR-1202 | *FRS2* | pita | -13.79 |
|  | hsa-miR-1202 | *LTBP2* | diana_microt | 0.872 |
|  | hsa-miR-1202 | *MAPK10* | pita | -13.23 |
|  | hsa-miR-1202 | *MAPT* | diana_microt | 0.825 |
|  | hsa-miR-1202 | *MMP2* | pita | -9.99 |
|  | hsa-miR-1202 | *NMNAT3* | pita | -9.86 |
|  | hsa-miR-1202 | *PEX2* | diana_microt | 0.804 |
|  | hsa-miR-1202 | *PIK3CG* | diana_microt | 0.907 |
|  | hsa-miR-1202 | *PTPN9* | pita | -10.51 |
|  | hsa-miR-1202 | *SALL1* | pita | -10.92 |
|  | hsa-miR-1202 | *SETD5* | pita | -10.35 |
|  | hsa-miR-1202 | *STAT3* | pita | -10.64 |
|  | hsa-miR-1202 | *THBS1* | pita | -10.27 |
|  | hsa-miR-1202 | *USH2A* | diana_microt | 0.832 |
|  | hsa-miR-1202 | *USP9X* | pita | -13 |
|  | hsa-miR-1202 | *VAV3* | pita | -11.81 |
|  | hsa-miR-1202 | *WT1* | pita | -11.6 |
|  | hsa-miR-1260b | *ARHGEF12* | pictar | 39.91 |
|  | hsa-miR-1260b | *ARL3* | pictar | 28.58 |
|  | hsa-miR-1260b | *CLRN1* | miranda | -1.0638 |
|  | hsa-miR-1260b | *CLRN1* | mirdb | 94.44 |
|  | hsa-miR-1260b | *CLRN1* | mirdb | 92.71 |
|  | hsa-miR-1260b | *CREB1* | pictar | 49.02 |
|  | hsa-miR-1260b | *CUX1* | pictar | 57.92 |
|  | hsa-miR-1260b | *ETS1* | pictar | 34.63 |
|  | hsa-miR-1260b | *PIK3R1* | pictar | 41.79 |
|  | hsa-miR-1260b | *PRPF3* | miranda | -1.03 |
|  | hsa-miR-1260b | *PRPF3* | miranda | -1.03 |
|  | hsa-miR-1260b | *TFAP2B* | pictar | 28.41 |
|  | hsa-miR-1260b | *XPR1* | diana_microt | 0.951 |
|  | hsa-miR-1260b | *XPR1* | pictar | 35.46 |
|  | hsa-miR-1260b | *XYLT1* | pictar | 40.05 |
|  | hsa-miR-184 | *ABO* | microcosm | 18.53 |
|  | hsa-miR-184 | *ABO* | microcosm | 18.53 |
|  | hsa-miR-184 | *COL1A1* | pita | -11.37 |
|  | hsa-miR-184 | *CYGB* | pita | -14.82 |
|  | hsa-miR-184 | *DAG1* | pita | -13.61 |
|  | hsa-miR-184 | *DAG1* | pita | -13.78 |
|  | hsa-miR-184 | *DBN1* | pita | -14.45 |
|  | hsa-miR-184 | *DOCK3* | pita | -14.59 |
|  | hsa-miR-184 | *DOK5* | pita | -10.44 |
|  | hsa-miR-184 | *ELN* | diana_microt | 0.856 |
|  | hsa-miR-184 | *JMJD1C* | diana_microt | 0.924 |
|  | hsa-miR-184 | *LRRC8A* | diana_microt | 0.838 |
|  | hsa-miR-184 | *MAPK9* | pita | -9.98 |
|  | hsa-miR-184 | *PLXNA2* | diana_microt | 0.865 |
|  | hsa-miR-184 | *PLXNA2* | pita | -17.72 |
|  | hsa-miR-184 | *PSD* | pita | -16.86 |
|  | hsa-miR-184 | *TBC1D20* | pita | -11.71 |
|  | hsa-miR-184 | *WNK1* | pita | -10.84 |
|  | hsa-miR-187-5p | *ABCA1* | diana_microt | 0.85 |
|  | hsa-miR-187-5p | *AGBL5* | miranda | -1.306 |
|  | hsa-miR-187-5p | *CADM2* | miranda | -1.096 |
|  | hsa-miR-187-5p | *CDKN1B* | mirdb | 94.87 |
|  | hsa-miR-187-5p | *CUX1* | elmmo | 0.724 |
|  | hsa-miR-187-5p | *CUX1* | miranda | -1.127 |
|  | hsa-miR-187-5p | *CUX1* | miranda | -1.127 |
|  | hsa-miR-187-5p | *CYP1B1* | diana_microt | 0.986 |
|  | hsa-miR-187-5p | *DCLK1* | diana_microt | 0.867 |
|  | hsa-miR-187-5p | *DCLK1* | elmmo | 0.661 |
|  | hsa-miR-187-5p | *ETS1* | diana_microt | 0.822 |
|  | hsa-miR-187-5p | *ETS1* | elmmo | 0.598 |
|  | hsa-miR-187-5p | *FRS2* | diana_microt | 0.915 |
|  | hsa-miR-187-5p | *FRS2* | elmmo | 0.677 |
|  | hsa-miR-187-5p | *FRS2* | mirdb | 93.13 |
|  | hsa-miR-187-5p | *NHS* | elmmo | 0.653 |
|  | hsa-miR-187-5p | *PDIK1L* | elmmo | 0.7 |
|  | hsa-miR-187-5p | *PDIK1L* | miranda | -1.079 |
|  | hsa-miR-187-5p | *PDIK1L* | mirdb | 89.642 |
|  | hsa-miR-187-5p | *PIK3CD* | elmmo | 0.534 |
|  | hsa-miR-187-5p | *SLC4A4* | diana_microt | 0.857 |
|  | hsa-miR-187-5p | *SLC4A4* | elmmo | 0.724 |
|  | hsa-miR-187-5p | *SLC4A4* | mirdb | 99.74 |
|  | hsa-miR-187-5p | *SLC4A4* | mirdb | 99.62 |
|  | hsa-miR-187-5p | *SLC4A4* | mirdb | 99.6 |
|  | hsa-miR-187-5p | *TGFBR1* | mirdb | 89.57 |
|  | hsa-miR-187-5p | *USP9X* | elmmo | 0.724 |
|  | hsa-miR-6515-3p | *PRDX6* | mirdb | 90.61 |
|  | hsa-miR-6515-3p | *SNCA* | mirdb | 94.42 |
|  | hsa-miR-6515-3p | *THSD7A* | mirdb | 97.83 |
|  | hsa-miR-6722-3p | *ARHGEF12* | pictar | 31.86 |
|  | hsa-miR-6722-3p | *BAK1* | mirdb | 91.17 |
|  | hsa-miR-6722-3p | *DPYSL5* | pictar | 62.74 |
|  | hsa-miR-6722-3p | *IGF1* | mirdb | 89.79 |
|  | hsa-miR-6722-3p | *IMPDH1* | pictar | 35.74 |
|  | hsa-miR-6722-3p | *MAPT* | pictar | 77.45 |
|  | hsa-miR-6722-3p | *SIGMAR1* | mirdb | 92.96 |
|  | hsa-miR-6722-3p | *SIGMAR1* | mirdb | 92.94 |
|  | hsa-miR-6722-3p | *SIGMAR1* | mirdb | 92.57 |
|  | hsa-miR-6722-3p | *SIGMAR1* | mirdb | 92.55 |
|  | hsa-miR-6722-3p | *SUFU* | pictar | 70.52 |
|  | hsa-miR-6722-3p | *TFAP2B* | pictar | 55.05 |


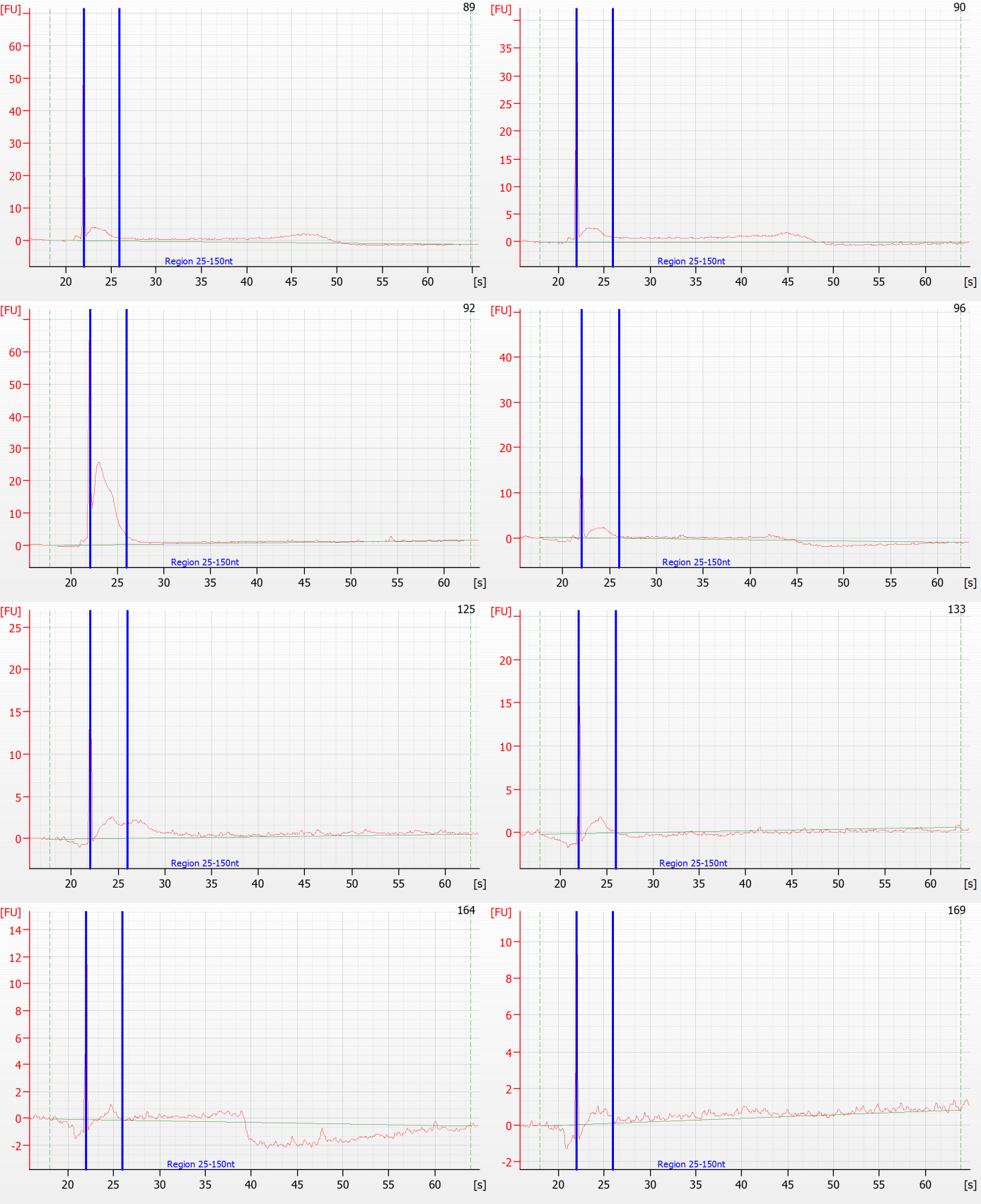


**Figure S1.** Electrophoregrams of aqueous humor samples analyzed on the Agilent Bioanalyzer 2100 using the RNA Pico Kit. The marked region corresponds to an RNA in the range of 25-150 nucleotides (miRNA fraction).
